# Behavioral weight-loss treatment plus motivational interviewing versus attention control: lessons learned from a randomized controlled trial

**DOI:** 10.1186/s13063-017-2094-1

**Published:** 2017-07-25

**Authors:** Erin L. Moss, Leah N. Tobin, Tavis S. Campbell, Kristin M. von Ranson

**Affiliations:** Department of Psychology, University of Calgary, 2500 University Dr. NW, Calgary, AB T2N 1N4 Canada

**Keywords:** Obesity, Motivational interviewing, Behavioral weight loss, Motivation, Treatment

## Abstract

**Background:**

Studies evaluating the benefit of adding motivational interviewing (MI) to behavioral weight-loss programs (BWLPs) have yielded mixed findings.

**Methods:**

The aims of this randomized controlled trial were to: (1) assess the efficacy of adding MI to a BWLP on weight loss and adherence among 135 individuals with overweight and obesity (77.8% female; mean BMI = 33.6 kg/m^2^) enrolled in a 12-week BWLP and (2) explore levels of importance, confidence, and readiness for change ratings.

**Results:**

Participants, who were randomized to receive two MI sessions or two attention control sessions, were assessed at baseline, the end of the BWLP, and 6 months post BWLP. Both groups decreased their weight from baseline to the end of the BWLP; however, there was no weight change in either group﻿ when measured between baseline and 6 months post BWLP. We observed no group differences in importance, confidence, and readiness for change after each session.

**Conclusions:**

We highlight some important lessons learned from the present trial that can be applied to MI + BWLP research. Participants may not have benefited from MI because they were already highly motivated to change, which highlights the importance of pretreatment assessment. Findings also suggest that treatment monitoring may help to enhance MI + BWLP efficacy by guiding a stepped-care approach that identifies individuals for whom additional MI sessions are needed, and when. A focus on refining elements of treatment remains an important direction.

**Trial registration:**

ClinicalTrials.gov, Identifier: NCT02649634. Retrospectively registered on 5 January 2016.

**Electronic supplementary material:**

The online version of this article (doi:10.1186/s13063-017-2094-1) contains supplementary material, which is available to authorized users.

## Background

Obesity rates are projected to continue rising globally, with 65 million more adults with obesity in the USA by 2030 [1]. Increased prevalence rates are concerning given obesity’s association with chronic illnesses and risk factors for death, including hypertension, stroke, type-2 diabetes, coronary heart disease, and dyslipidemia [2, 3]. Obesity, which places a large economic burden on health care systems due to medical complications [1], can be addressed through public health and behavioral interventions. Research focused on improving behavioral treatment outcomes for obesity is greatly needed.

In this study, we investigated the effect of administering motivational interviewing (MI) to adults with overweight and obesity who had already voluntarily enrolled in a behavioral weight-loss program (BWLP). MI is a counseling strategy that aims to assist and motivate individuals in moving towards behavioral change. BWLPs attempt to address environmental causes of obesity by increasing energy expenditure and decreasing energy intake through behavioral changes, and are usually the first line of weight-loss treatment [4]. A BWLP typically includes dietary intervention, physical activity change, and behavioral self-management [5, 6].

In addition to yielding small to moderate weight reductions in populations with obesity [7–9], randomized controlled evaluations of BWLPs have also demonstrated dietary improvements and increases in levels of physical activity [10]. However, there is room for improvement with current BWLPs. Participants in these programs typically lose approximately 5 to 10% of their weight from baseline [3, 11, 12]. Weight loss appears to plateau at the 10% mark [6], despite considerable attempts to improve BWLP outcomes [13, 14]. Two major issues in BWLPs that may limit the effectiveness of treatment are high rates of attrition and poor adherence to the treatment program [15, 16]. Given that MI focuses on assisting and motivating individuals towards behavioral change, it might improve weight-loss treatment outcomes by enhancing treatment engagement and improving adherence to dietary and exercise requirements [17].

A research base examining the integration of MI with BWLP is growing. A meta-analysis examining the effects of MI on weight loss found a moderate effect of MI on body mass using a random effects model (standardized mean difference = −0.51) [18]. On average, MI enhanced weight loss by 1.47 kg relative to control conditions, and studies that included weight loss as a primary outcome and/or employed MI as an adjunct to a BWLP demonstrated the best results. However, of the five studies that examined the effect of adding MI to a BWLP in this meta-analysis, three failed to find any weight-loss benefit for MI [19–21]. Proposed reasons for these null findings included socioeconomic barriers to change faced by certain samples [19], inadequate sample size, lack of or uncertain treatment fidelity, and inadequate long-term follow-up [20, 21]. Another study investigating MI as an adjunct to an Internet-based BWLP also failed to find an effect of MI on weight loss [22]. However, these authors [22] reported that among individuals with high baseline levels of controlled motivation (i.e., external pressure to change), those receiving MI showed greater weight loss than those receiving BWLP only, suggesting that baseline levels of motivation might help to identify who will benefit most from adding MI to a BWLP. Overall, evidence is mixed regarding with which individuals MI is an effective adjunct to BWLPs. There is a need for methodologically rigorous research that maximizes the efficacy of MI adjunct to BWLPs.

The constructs of importance, confidence, and readiness for change are considered critical elements in the conceptualization of motivation [23]. Reviews suggest that confidence and readiness to change, in particular, are modifiable aspects which can help prevent excessive gestational weight gain [24, 25]. While other fields incorporating MI with existing treatments, including substance use [26] and gestational weight gain [27], have studied these constructs, the constructs have been largely unexplored in the MI + BWLP literature. It is unclear if MI, when added to a BWLP, affects these dimensions of change, or if they help to distinguish individuals who might benefit from MI.

The aim of the current study was to examine the effect of adding two MI sessions to a BWLP among adults seeking weight loss. We modeled our methods on the study that has demonstrated the largest effect to date of MI + BWLP on weight loss, namely West and colleagues’ [28] study of women with obesity and type-2 diabetes which included an attention control condition, assessed treatment fidelity, and tested a large sample size. Additionally, we explored individual differences in factors related to motivation for change, including ratings of importance of, readiness for, and confidence for, change.

We hypothesized that participants in a BWLP who also received MI would reduce body weight by end of treatment and at a 6-month follow-up to a greater degree than controls. In addition, given that MI has shown promise for improving adherence to BWLPs [21], we hypothesized that the MI group would show greater adherence to the BWLP than the control group. Given that MI specifically aims to enhance self-efficacy and motivation, an exploratory hypothesis was that the MI group would report greater importance, confidence, and readiness for change ratings immediately after both MI sessions relative to the control group.

## Methods

This randomized controlled trial investigated the effects of an MI intervention on body mass and related outcomes in adults with overweight and obesity who had voluntarily self-enrolled and paid for TrymGym, a 12-week, 24-session BWLP at the University of Calgary. An additional file shows the Consolidated Standards of Reporting Trials (CONSORT) 2010 Checklist for randomized trials (see Additional file 1). Calgary is a western Canadian city of 1.2 million people, 30.1% of whom identify themselves as visible minorities [29, 30]. Eligible participants were randomized to receive either two MI intervention sessions or two attention-control interviews in addition to the BWLP, with an allocation ratio of 1:1. The estimated sample size needed in each condition was 45, determined by an a-priori power estimate for two groups with alpha set at 0.05, power at 0.80, and anticipating a medium effect size [31]. We chose a medium effect size on the basis of a meta-analysis of MI for weight loss available at the time of study design that reported an overall medium effect of .53 [32]. To account for anticipated attrition of 32% [15], we recruited 135 participants in total.

### Participants

Recruitment took place from September 2007 to May 2009. Once a sample size of 135 participants had been achieved, recruitment was ended. Entire study duration, including all follow-up assessments, was from September 2007 to January 2010. Participants were eligible if they were 18 years or older and in the Body Mass Index (BMI) range of overweight or obesity (BMI ≥25 kg/m^2^). Exclusion criteria included pregnancy or intention of becoming pregnant within 9 months, health issues that would preclude participation in physical activity, or concurrent involvement in another weight-loss program.

### Procedure

Research personnel informed all TrymGym participants about the study at the initial group intake assessments, just prior to the commencement of the formal BWLP. Individuals who expressed interest in participating were contacted by phone and screened for eligibility by a research assistant. If eligible, an appointment was made for the first MI/control session within the first 2 weeks of the BWLP. Participants were asked to complete change rating questionnaires prior to this interview (i.e., importance, readiness, and confidence for change ratings), and randomization occurred immediately prior to this interview. After the first interview, participants completed a second MI/control session approximately during the 12th week of the program. Finally, participants were contacted approximately 5 months following program completion to schedule a 6-month in-person follow-up assessment.

### Treatment

#### Behavioral weight-loss program

TrymGym was established in 1973 at a large medical-doctoral university in Calgary and over 10,000 participants have completed the program. This BWLP emphasizes gradual, sustainable weight loss and lifestyle changes, and is delivered by a team of health care practitioners including dietitians, kinesiologists, and fitness instructors via both classroom sessions and exercise sessions. Specifically, the program consists of three core components: (1) Nutrition: individualized guidelines for healthy eating, based on the Canada Food Guide [33], were developed for each participant, (2) Physical activity: group exercise classes focused on fat loss, strength training, and development of endurance and flexibility, and (3) Behavior change: behavioral strategies including self-monitoring, goal-setting, and formulating action plans to achieve goals were taught in classroom sessions.

#### Motivational interviewing intervention

The semi-structured MI protocol was a 45-min intervention developed by the first author based on general MI principles and guidelines [23], MI strategies specific to health care practice [34], and MI principles for obesity treatment [35]. The MI protocol included the following components: (1) eliciting concerns about weight, (2) exploring ambivalence, (3) assessing importance and confidence for change, (4) writing a decisional balance, (5) bolstering self-efficacy, (6) looking towards the future, and (8) eliciting ideas for possible changes participant could make to work towards weight loss. The protocol for both MI sessions consisted of similar components.

#### Attention control intervention

The attention control interview was a semi-structured interview addressing health history, weight history, diet history, and dietary and physical activity habits. Most questions were drawn from the TrymGym intake application. It was designed to be structurally equivalent to the MI session in length of session, timing of sessions, and treatment modality. The goal was to provide a pseudo-intervention that controlled for factors common to attending treatment (e.g., attending treatment sessions, having personal contact with a therapist, discussing weight-related issues).

### Therapist training and supervision

The first author delivered all of the MI and control sessions, both for practical reasons and to help control for possible therapist effects. Therapist training consisted of over 20 h of readings [23, 34], video [36], role play, discussions of MI principles and strategies, and a total of 8 days of workshop training facilitated by members of the Motivational Interviewing Network of Trainers. Ongoing supervision was provided by a doctoral-level clinical psychologist (KMvR) throughout.

### Randomization

Immediately prior to the first interview, the first author randomly allocated participants to either the MI or control group with Minim, a computerized randomization program (http://www-users.york.ac.uk/~mb55/guide/minim.htm). Minimization was used to allow for the balancing of groups on participant gender, the only covariate upon which we elected to ensure group equivalence. Participants were blind to treatment group assignment.

### Treatment integrity

With participants’ consent, all sessions (both MI and control) were audio-recorded for quality assurance purposes. Of recorded sessions, 10% from the first interview and 10% from the second interview were randomly selected for treatment integrity assessment. Random 20-min segments of these tapes were coded by a doctoral-level clinical psychologist with extensive MI training and experience using the Motivational Interviewing Treatment Integrity (MITI) system [37], described below. The coder (TSC) was not involved in delivering any of the MI sessions.

### Assessment measures

Weight measurements were collected by TrymGym staff members (at baseline and the end of BWLP) or a research assistant (6-month follow-up), who were blind to participants’ treatment condition. Change ratings were collected online prior to the first interview, and in-person immediately after the first and second interviews.

#### Anthropometric measurements

Weight was measured to the nearest 0.1 kg, using a balance beam scale at baseline and at the end of the program and with a portable digital scale (Tanita BWB-800S) at the 6-month follow-up assessment.

#### Treatment adherence

Treatment adherence was assessed via attendance (present versus absent) at each of 24 BWLP group meetings and exercise classes.

#### Motivational Interviewing Treatment Integrity system

The MITI system [37], which was used to assess MI treatment fidelity, is composed of two global scores, empathy and MI spirit, which are rated on a 7-point scale to characterize an entire interaction. The MITI also consists of five behavior counts: (1) giving information (GI), (2) open-ended (OQ)/close-ended (CQ) questions, (3) simple (RS)/complex (RC) reflections, (4) MI-adherent behaviors (MIA; e.g., asking permission, affirming, emphasizing control), and (5) MI-nonadherent behaviors (MINA; e.g., advising, confronting, directing). Suggested guidelines indicative of MI beginning proficiency are: global scores ≥5, reflection to question ratio >1, percent OQ >50%, RC >40%, and percent MIA >90%.

#### Importance, confidence, and readiness for change

Three separate questions inquired about participants’ importance, confidence, and readiness for change. Each construct was assessed via a Visual Analogue Scale, e.g., “On a scale of 0–10, how confident are you that you can, or could, lose weight?” “On a scale of 0–10, how ready are you to lose weight?” “On a scale of 0–10, how important is it to you to lose weight?” [23]. Lower scores reflect lower levels of importance, confidence, and readiness for change. These scales have been used to measure motivation change previously [38, 39].

### Data analysis

#### Sample characteristics, baseline analyses, and randomization

To confirm that participants randomized to the MI and control conditions were comparable, all demographic characteristics and baseline scores on self-report questionnaires were analyzed using independent samples *t* tests or *χ* ^*2*^ tests.

#### Treatment dropout rates and study assessment dropout rates

To examine whether attrition rates differed between the MI and control groups a *χ* ^*2*^ test was performed. To investigate whether demographic characteristics and baseline scores of participants who completed the study were comparable to noncompleters independent samples *t* tests and *χ* ^*2*^ tests were performed.

#### Primary hypothesis

Linear mixed modeling (LMM), also known as mixed-effects modeling, was used to examine the primary hypothesis. One of the most significant benefits of LMM is the capacity of this analysis to flexibly handle missing data [40]. The observed data showed nonlinear trends in the outcome variable, so longitudinal trends for the outcome variable were modeled using time as a categorical variable; specifically time 2 (period from baseline to the end of the BWLP) and time 3 (period from baseline to 6-month follow-up). Initially, the potential interaction between group and time was examined by entering the following variables: group, time, baseline value of outcome variable, and the interaction term between time and group. In this model, the interaction term determined if the intervention effect varied between groups. If the interaction term was nonsignificant, the model was refitted with only the main effect terms. For the main effect model, if the “time” variable was significant, contrasts were specified to examine change relative to baseline. Alpha was set at .05. The structure of the repeated measures was modeled by including intercept as a random effect at the subject level. Model estimates were obtained using maximum likelihood estimation (ML) implemented with the linear mixed models module of SPSS. In the analysis, a variance-components variance-covariance matrix was estimated. Effect sizes for treatment effects were calculated from LMM estimates using the equation [41]:

$$ {d}_{\mathrm{GMA}\hbox{-} \mathrm{RAW}} = \mathrm{estimated}\ \mathrm{coefficient}\left(\mathrm{time}\right)/{\mathrm{SD}}_{\mathrm{RAW}} $$

#### Secondary hypothesis

Treatment adherence was analyzed via an independent samples *t* test comparing the treatment groups on mean number of BWLP sessions missed.

#### Exploratory hypothesis

Cohen’s *d* effect sizes, calculated with mean change scores and pooled standard deviations, were examined to determine whether the two groups differed on importance, readiness, and confidence for change scores immediately following both intervention sessions.

## Results

### Sample characteristics and baseline analyses

A total of 159 participants were assessed for study eligibility, and 105 women and 30 men participated in this study. Of the 24 individuals excluded from the study, two were ineligible due to health concerns precluding participation in physical activity, 17 declined involvement due to the time commitment required, and five were unable to be contacted. At baseline, participants’ mean age was 45.16 years (*SD* = 11.30), mean BMI was 33.58 kg/m^2^ (*SD* = 6.26), and mean weight was 92.78 kg (*SD* = 20.57). The majority of participants were Caucasian (93.3%), married or in a common-law relationship (71.1%), employed full-time (68.1%), had an annual family income exceeding CAD80,000 (53.7%), and had completed university (63.7%). No demographic or baseline differences were found between participants in the MI and control groups (see Additional file 2). There were no interactions or main effects for gender with any of the primary or secondary outcome variables (all *p* > .17).

### Treatment dropout rates and study assessment dropout rates

**Fig. 1:**
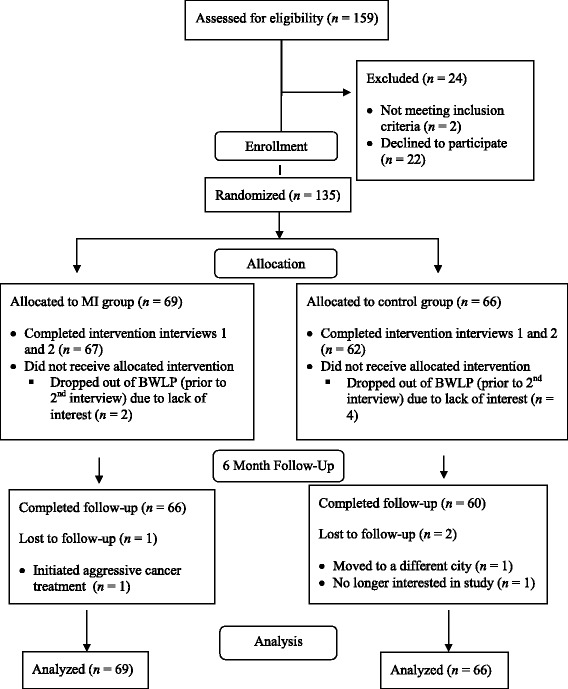
Participant flow diagram

One hundred and twenty-six participants completed their allocated intervention as well as the 6-month follow-up assessment (93.33%). Dropout rates for the MI group (4.35%) and control group (9.09%) did not differ, *χ* ^*2*^ (1, *N* = 135) = 1.22, *p* = .32. See Fig. 1 for the CONSORT participant flow diagram. See CONSORT Checklist for details. No demographic (all *p* > .06) or outcome variables (all *p* > .31) differed between study completers and dropouts (see Additional files 3 and 4).

### Treatment fidelity

All MI scores met the suggested guidelines for proficiency, whereas control group scores were well below the suggested guidelines for MI proficiency [37] (see Table 1).

**Table 1:** MI and control group fidelity ratings on the Motivational Interviewing Treatment Integrity (MITI) system

	MI group (*n =* 69)			Control group (*n =* 66)		
Scores	Means	+ SD	Ranges	Means	+ SD	Ranges
Empathy	5.50	.44	5–6	1.50	.34	1–2
MI spirit	5.50	.44	5–6	1.50	.34	1–2
Giving information	0.14	.36	0–1	0.43	.16	0–4
Open-ended questions	11.0	3.8	5–20	5.57	3.30	1–11
Close-ended questions	0.43	1.09	0–4	28.93	15.53	10–62
Simple reflections	11.36	3.43	7–18	5.50	3.30	0–10
Complex reflections	11.07	4.25	5–21	0	0	0–0
MI-adherent behaviors	4.86	2.96	1–11	0.07	.27	0–1
MI-nonadherent behaviors	0	0	0–0	0	0	0–0
Reflection-question ratio^a^	2.11	.81	1.14–4.60	0.18	.12	0– .40
	Percentages	+ SD	Ranges	Percentages	+ SD	Ranges
Percent open-ended questions^b^	96.97	7.76	71–100	17.81	10.73	3–35
Percent complex reflections^c^	48.77	8.40	38–65	0	0	0–0
Percent MI-adherent behaviors^d^	100	0	100–100	100	0	100–100

*MI* motivational interviewing, *SD* standard deviation

^a^Reflection-question ratio = total reflections/(close-ended questions [CQ] + open-ended questions [OQ])

^b^Percent open-ended questions = OQ/(OQ + CQ questions)

^c^Percent complex reflections = complex reflections/total reflections

^d^Percent MI-adherent behaviors = MI-adherent behaviors (MIA)/MIA + MI-nonadherent behaviors

### Primary hypothesis: weight

The interaction model was not significant, indicating no effect of MI over time on outcome. For the main effects model of body weight, group was not significant, *F*(1, 139.26) = 1.14, *p* = .29, but time was significant, *F*(2, 261.55) = 22.70, *p* < .001. For both groups, body weight decreased from baseline to the end of the BWLP, *B* = −2.55, *SE* = .39; *t*(262.50) = −6.54, *p* < .001 (CI = −3.31, −1.78), but body weight did not change from baseline to the 6-month follow-up, *B* = −.66, *SE* = .39; *t*(261.53) = −1.70, *p* = .09 (CI = −1.42, .10) (see Table 2).

**Table 2:** Comparison of weight outcomes over time for MI versus control groups

	Baseline		End of BWLP					6-month follow-up				
Outcome measure			Unadjusted		Adjusted^a^		Effect size	Unadjusted		Adjusted^a^		Effect size
	*M*	*SD*	*M*	*SD*	*M*	*SE*		*M*	*SD*	*M*	*SE*	
Weight (kg)												
MI Group	95.11	21.45	91.32	20.78	89.02	.43	+.045	95.08	22.83	91.26	.42	+.009
Control group	90.34	19.46	85.77	16.65	90.09	.45		87.47	17.29	91.59	.45	

*MI* motivational interviewing, *BWLP* behavioral weight-loss program

^a^Mean values calculated with baseline value as covariate. LMM effect size calculated as d_GMA-RAW_ = estimated coefficient(time)/SD_RAW_ [39]. (+) effect size favors MI group

### Secondary hypothesis: treatment adherence

The mean number of missed BWLP sessions was compared across groups to assess treatment adherence. The MI group missed a mean of 3.71 (*SD* = 4.12; range = 0 to 13) sessions, and the control group missed a mean of 4.07 (*SD* = 3.60; range = 0 to 14) sessions. These means did not differ between groups, *t*(102) = −.46, *p* = .65.

### Exploratory hypothesis: importance, readiness, and confidence for change ratings

Following both interviews, standardized effect sizes for change ratings were generally below Cohen’s [42] minimum threshold for a small effect (*d* = 0.20), suggesting no group differences (see Table 3).

**Table 3:** Comparison of motivational interviewing (MI) and control group on weight change ratings immediately following interventions

	MI Baseline (*N* = 69)	Control Baseline (*N* = 66)	MI after Interview 1 (*N* = 69)	Control after Interview 1 (*N* = 66)	MI after Interview 2 (*N* = 67)	Control after Interview 2 (*N* = 62)	*d* after Interview 1	*d* after Interview 2
Variable	*M* (*SD*)	*M* (*SD*)	*M* (*SD*)	*M* (*SD*)	*M* (*SD*)	*M* (*SD*)	*M* (*SD*)	*M* (*SD*)
Importance of change	8.88 (1.25)	9.00 (1.35)	8.67 (1.30)	8.73 (1.60)	8.52 (1.23)	8.88 (1.54)	.05	−.18
Readiness for change	8.70 (1.34)	8.80 (1.39)	8.64 (1.32)	8.65 (1.22)	8.70 (1.14)	8.86 (1.33)	.07	−.04
Confidence for change	7.79 (1.76)	8.14 (1.63)	7.85 (1.54)	8.06 (1.38)	8.25 (1.20)	8.39 (1.26)	.08	.12

Note. Between-group effect size (Cohen’s *d*) calculated with mean change scores and pooled standard deviations. (+) effect size favors MI group; (−) effect size favors control group

## Discussion

These results indicate that participants’ weight decreased from baseline to the end of the BWLP in both the MI + BWLP and BWLP-only (control) groups, but the addition of MI did not enhance weight loss. However, no change in participants’ body weight in either group was observed between baseline and 6-month follow-up, indicating that BWLP effects were not sustained, possibly due in part to the brevity of the 12-week BWLP. Supporting this interpretation, a meta-analysis found that BWLP durations longer than 6 months generally yielded more weight loss relative to briefer BWLPs [18], and this increased initial weight loss may delay potential weight regain. Furthermore, results showed that participants who had received MI were no more likely to have attended more treatment sessions than control participants. Additionally, self-report ratings of importance, readiness, and confidence for change did not differ between groups after the first or second MI/control session, indicating that the MI also had no effect on these critical elements in the conceptualization of motivation. Although we assessed the effects of the MI + BWLP intervention on several additional indices of health outcomes (i.e., BMI, physical activity, dietary behavior, blood pressure, and eating disorder psychopathology), we found similar results as for the weight, adherence, and change rating outcomes described above. Overall, the addition of MI to the BWLP did not change outcomes on any of these measures (see Additional file 5 for a detailed description of these additional outcome measures and results).

In sum, we observed no indication that MI + BWLP increased weight loss or that it improved related motivational constructs, as effect sizes fell below the minimum threshold for a small effect. Together with previous null findings, our results suggest that, at best, MI is not consistently effective in improving weight-loss outcomes in BWLPs. Research to date – including the present null findings – could prove informative for devising future investigations seeking to enhance weight loss in BWLPs. With the aim of improving future randomized controlled trials for MI + BWLP, we now highlight important lessons learned in the present trial.

### Lesson 1: use a stepped-care approach

In stepped-care models, additional treatment sessions are provided to those who need them. We administered MI in a standard fashion to participants, using a predetermined MI protocol, and did not tailor the components of MI to match each participant’s level of readiness to change. Contrary to our expectations, participants receiving MI did not report greater importance of change, readiness for change, or confidence for change following each MI session than control participants, suggesting no discernible effect of MI on these measures of motivation. Notably, as all three ratings were high at baseline (see Additional file 4) we may have encountered a ceiling effect. Assessment of these specific aspects of motivation at baseline and throughout treatment could have been used to help guide a stepped-care approach to treatment. Perhaps if we had followed Miller and Rollnick’s [23] suggestion to adapt MI to the client’s stage of readiness to change, we would have increased the effectiveness of the MI + BWLP. For example, Carels et al. [43] used a stepped-care approach in which they identified individuals who had failed to meet weight-loss goals during the course of a BWLP and administered additional 45- to 60-min individual sessions until weight-loss goals were achieved. This approach was successful: the MI group lost more weight and increased planned physical activity more than a control group with no additional treatment. It is likely that selecting those individuals most likely to benefit from motivational enhancement and adapting treatment dose to meet these clients’ needs contributed to effective outcomes in this study.

In addition, if administered over the follow-up period, we speculate that MI might help individuals to maintain changes at a point when motivation might be more likely to wane. Future research might investigate whether such an approach improves weight-loss maintenance.

### Lesson 2: assess pretreatment motivation to determine MI necessity for the sample

It is important to focus on assessing differences in motivation for the sample of interest prior to conducting MI + BWLP research. Recently, attention has been directed to specific elements of methodological quality in clinical trials, such as the Obesity-Related Behavioral Intervention Trials (ORBIT) model [44, 45]. Rather than assume universal impact of behavioral interventions, such as MI, on weight loss, it is preferable to determine the need for tailoring of interventions to specific populations [44, 45]. For example, although in prior MI + BWLP research the BWLP was provided at no cost to participants; participants in the present study had sufficient motivation prior to treatment to have paid to participate in the BWLP. Given that MI may be differentially effective depending on one’s initial motivation to change, stand-alone BWLPs may be sufficient for individuals with higher initial levels of motivation without the addition of MI. However, many individuals with obesity in the community who might benefit from a BWLP may not have the pretreatment motivational levels to seek out these effective programs. An alternative strategy would be to use MI to target the motivation of these individuals to initially attend a BWLP. Overall, comprehensive assessment of a sample’s pretreatment motivation for change appears to be important in future research. Assessing a sample’s pretreatment motivation may assist with further refining our understanding of whom in particular may benefit from MI + BWLP.

### Lesson 3: refine the definition of the motivation construct – consider assessing both controlled and autonomous motivation

There has been increasing interest in the application of Self-determination Theory (SDT) [46, 47] to MI as a means to better operationalize the construct of motivation [48–50]. Applications of this theory propose that there is an important distinction between a client’s level of *controlled* (i.e., extrinsic) versus *autonomous* (i.e., intrinsic) motivation [51, 52]. It is suggested that greater autonomous motivation is associated with greater odds of success for behavioral change, whereas controlled motivation is associated with lower odds of success [22, 53]. Consequently, MI may be best suited for individuals with high controlled motivation and less suitable for those with high autonomous motivation (e.g., those who choose to enroll in a weight-loss program for personal reasons and do not feel compelled by external factors to lose weight). For example, in a study that failed to find a benefit of adding MI to a BWLP [22], post hoc analyses found that the effect of MI on weight-loss outcomes was moderated by level of baseline controlled motivation. Specifically, among individuals with high controlled motivation, the MI group experienced greater weight loss than the control group. Several recent studies have used SDT and assessment of autonomous and controlled motivation as a core tenet of weight-loss interventions with promising results [54–56], and currently components of SDT are being integrated with MI to develop a tailored intervention related to the promotion of physical activity (e.g., Moreau et al. [57]). Thus, distinguishing controlled and autonomous motivation in assessment in MI + BWLP studies may help refine the conceptualization of the construct of motivation and better guide MI + BWLP treatment.

### Strengths and limitations

This MI + BWLP study had several strengths. Specifically, we assessed fidelity, incorporated an attention control group, and included a longer-term follow-up. Our sample size was large, we included both women and men, and we did not limit recruitment to individuals with a specific medical concern. Additionally, we assessed dimensions theoretically related to motivation for change and used a sophisticated statistical procedure to handle missing data.

However, this study also had several limitations. First, given our participants’ high baseline self-reported ratings of importance, readiness, and confidence for change, we speculate that they may have already been high in autonomous motivation prior to treatment. Therefore, this sample may not have been well-suited to benefit from the addition of MI to a BWLP. Second, the MI dose provided in this study may have been inadequate to improve weight-loss outcomes. Compared to the average dosage in MI for weight-loss research, in hindsight the MI intervention we provided was minimal. The analysis by Armstrong et al. [18] showed that, of studies that had investigated an effect on weight of MI in conjunction with a BWLP, those that demonstrated a significant effect of MI had offered five or more sessions. The fact that the present study included only two sessions of MI influences the conclusions that can be drawn regarding the efficacy of MI + BWLP for weight loss. Third, we relied on self-report outcome data for the exploratory outcome measures (i.e., change ratings), which may be subject to self-reporting biases. Fourth, the sample was composed largely of White, higher-socioeconomic-status individuals, which limits the generalizability of findings to other populations.

## Conclusions

Although MI may have promise in the treatment of obesity, further research to isolate specific elements and patient characteristics that contribute to improved MI outcomes is essential. We encourage future investigators to: (1) assess motivation throughout treatment to enable provision of a stepped-care approach, (2) assess pretreatment motivation to determine whether MI is appropriate for the population of interest, and (3) refine assessment of the motivation construct to include assessments of both controlled and autonomous motivation, before and throughout treatment. Carefully controlled trials that incorporate recommendations of the ORBIT model in their design are needed to advance knowledge about maximizing efficacy of weight loss. For example, refining MI + BWLP treatments by developing “adaptive treatments with tailoring for special population subgroups or for differential response to treatment” [45; p. 976] is needed. It is not yet clear what minimum dose of MI is needed to improve BWLP outcomes, but dose may need to vary according to sample, individual characteristics, and ongoing assessment of motivation. Continued efforts targeted towards careful, methodological improvement of existing interventions, such as MI+ BWLP, and development of new forms of treatment remain important goals for effective obesity treatment.

## Additional files

Additional file 1: Table S1. CONSORT 2010 Checklist of information to include when reporting a randomized trial. (DOCX 19 kb)

Additional file 2: Table S2. Comparison between motivational interviewing (MI) and control groups on demographic characteristics at baseline. (DOCX 17 kb)

Additional file 3: Table S3. Comparison of baseline demographics in study completers and dropouts. (DOCX 17 kb)

Additional file 4: Table S4. Comparison between study completers and dropouts on primary and exploratory outcome variables at baseline. (DOCX 16 kb)

Additional file 5: Additional secondary outcome variables and associated results. A list of additional secondary outcome variables used to provide additional indicators of health, and their associated data analyses and results. (DOCX 42 kb)

## Abbreviations

BMI: Body Mass Index
BWLP: Behavioral weight-loss program
CQ: Close-ended questions
GI: Giving information
LMM: Linear mixed modeling
MI: Motivational interviewing
MIA: Motivational interviewing-adherent behaviors
MINA: Motivational interviewing nonadherent behaviors
MITI: Motivational Interviewing Treatment Integrity system
ML: Maximum likelihood estimation
OQ: Open-ended questions
ORBIT: Obesity-Related Behavioral Intervention Trials
RC: Complex reflections
RS: Simple reflections
SDT: Self-determination Theory
