# Supplementary material for: Behavioral weight-loss treatment plus motivational interviewing versus attention control: lessons learned from a randomized controlled trial
Source: Trials. 2017 Jul 25;18:351. doi: 10.1186/s13063-017-2094-1 (PMC5526285; doi:10.1186/s13063-017-2094-1)
Supplement: Supplementary file 1 — CONSORT 2010 Checklist of information to include when reporting a randomized trial. (DOCX 19 kb) [file 13063_2017_2094_MOESM1_ESM.docx]

Table S1

*CONSORT 2010 Checklist of Information to Include when Reporting a Randomized Trial*

| Selection/topic | Item number | Checklist item | Reported on page number |
| --- | --- | --- | --- |
| **Title and abstract** | 1a | Identification as a randomized trial in the title | 1 |
|  | 1b | Structured summary of trial design, methods, results, and conclusions | 2 |
| **Introduction** |  |  |  |
| Background and objectives | 2a | Scientific background and explanation of rationale | 2-5 |
|  | 2b | Specific objectives or hypotheses | 5 |
| **Methods** |  |  |  |
| Trial design | 3a | Description of trial design (such as parallel, factorial) including allocation ratio | 5-6 |
|  | 3b | Important changes to methods after trial commencement (such as eligibility criteria), with reasons | n/a |
| Participants | 4a | Eligibility criteria for participants | 6 |
|  | 4b | Settings and locations where the data were collected | 6-7 |
| Interventions | 5 | The interventions for each group with sufficient details to allow replication, including how and when they were actually administered | 6-7 |
| Outcomes | 6a | Completely defined pre-specified primary and secondary outcome measures, including how and when they were assessed | 6-9 |
|  | 6b | Any changes to trial outcomes after the trial commenced, with reasons | n/a |
| Sample size | 7a | How sample size was determined | 6 |
|  | 7b | When applicable, explanation of any interim analyses and stopping guidelines | n/a |
| Randomization: |  |  |  |
| Sequence | 8a | Method used to generate the random allocation sequence | 8 |
| generation | 8b | Type of randomization; details of any restriction | 8 |
| Allocation  concealment  mechanism | 9 | Mechanism used to implement the random allocation sequence (such as sequentially numbered containers), describing any steps taken to conceal the sequence until interventions were assigned | 8 |
| Implementation | 10 | Who generated the random allocation sequence, who enrolled participants, and who assigned participants to interventions | 6, 8 |
| Blinding | 11a | If done, who was blinded after assignment to interventions (for example, participants, care providers, those assessing outcomes) and how | 8-9 |
|  | 11b | If relevant, description of the similarity of interventions | 7 |
| **Results** |  |  |  |
| Participant flow | 13a | For each group, the numbers of participants who were randomly assigned, received intended treatment, and were analysed for the primary outcome | Figure 1 |
|  | 13b | For each group, losses and exclusions after randomization, together with reasons | Figure 1 |
| Recruitment | 14a | Dates defining the periods of recruitment and follow-up | 6 |
|  | 14b | Why the trial ended or was stopped | 6 |
| Baseline data | 15 | A table showing baseline demographics and clinical characteristics for each group | Supplementary Table 2, Table 2, Table 3 |
| Numbers analysed | 16 | For each group, numbers of participants (denominator) included in each analysis and whether the analysis was by original assigned groups | Figure 1  Tables 1, 3 |
| Outcomes and estimation | 17a | For each primary and secondary outcome, results for each group, and the estimated effect size and its precision (such as 95% confidence interval) | 12-13  Tables 2, 3 |
|  | 17b | For binary outcomes, presentation of both absolute and relative effect sizes is recommended | n/a |
| Ancillary analyses | 18 | Results of any other analyses performed, including subgroup analyses and adjusted analyses, distinguishing pre-specified from exploratory | 11-12,  Supplementary Tables 3 & 4 |
| Harms | 19 | All important harms or unintended effects in each group | n/a |
| **Discussion** |  |  |  |
| Limitations | 20 | Trial limitations, addressing sources of potential bias, imprecision, and if relevant, multiplicity of analyses | 16 |
| Generalisability | 21 | Generalisability (external validity, applicability) of the trial findings | 13-17 |
| Interpretation | 22 | Interpretation consistent with results, balancing benefits and harms, and considering other relevant evidence | 13-16 |
