# Supplementary material for: Behavioral weight-loss treatment plus motivational interviewing versus attention control: lessons learned from a randomized controlled trial
Source: Trials. 2017 Jul 25;18:351. doi: 10.1186/s13063-017-2094-1 (PMC5526285; doi:10.1186/s13063-017-2094-1)
Supplement: Supplementary file 2 — Comparison between motivational interviewing (MI) and control groups on demographic characteristics at baseline. (DOCX 17 kb) [file 13063_2017_2094_MOESM2_ESM.docx]

Table S2

*Comparison between MI and Control Groups on Demographic Characteristics at Baseline*

|  | Means (+ SD) | | *t* | *df* | | *p* |
| --- | --- | --- | --- | --- | --- | --- |
| Characteristics | MI  (*n=* 69) | Control  (*n=* 66) |  |  | |  |
| Age (years) | 45.56 (9.78) | 44.67 (12.91) | .46 | 132 | | .65 |
|  | % | | *χ^2^* | | *df* | *p* |
| Gender |  |  | .48 | | 1 | .49 |
| Female | 75.36 | 80.30 |  | |  |  |
| Male | 24.64 | 19.70 |  | |  |  |
| Ethnicity^a^ |  |  | 1.38 | | 1 | .31 |
| Caucasian | 95.77 | 90.63 |  | |  |  |
| Other^b^ | 4.29 | 9.38 |  | |  |  |
| Marital status^a^ |  |  | 2.60 | | 2 | .27 |
| Married/Common-law | 76.81 | 65.15 |  | |  |  |
| Divorced/Separated | 8.70 | 16.67 |  | |  |  |
| Never Married | 14.49 | 18.18 |  | |  |  |
| Education Level^a^ |  |  | .27 | | 3 | .98 |
| Completed High School | 13.04 | 10.60 |  | |  |  |
| Some University | 23.19 | 25.76 |  | |  |  |
| Completed University | 46.38 | 46.97 |  | |  |  |
| Completed Graduate School | 17.39 | 16.67 |  | |  |  |
| Annual Family Income^a^  ($) |  |  | 7.04 | | 3 | .13 |
| 20,000-39,999 | 4.17 | 20.93 |  | |  |  |
| 40,000-59,999 | 12.50 | 9.30 |  | |  |  |
| 60,000-79,999 | 12.50 | 13.95 |  | |  |  |
| >80,000 | 70.83 | 55.82 |  | |  |  |
| Employment Status^a^ |  |  | 7.94 | | 4 | .09 |
| Full time | 75.36 | 60.61 |  | |  |  |
| Part time | 10.14 | 15.15 |  | |  |  |
| Homemaker | 8.70 | 4.54 |  | |  |  |
| Retired | 4.35 | 10.61 |  | |  |  |
| Unemployed | 1.45 | 9.09 |  | |  |  |

*Note.* ^a^ some cells had an expected count less than 5, so a Fisher’s exact significance test

was selected. ^b^ due to low numbers in the different ethnicity cells, they were collapsed in the

“other group,” which consists of Asian, East Indian, and Hispanic.
