# Supplementary material for: Behavioral weight-loss treatment plus motivational interviewing versus attention control: lessons learned from a randomized controlled trial
Source: Trials. 2017 Jul 25;18:351. doi: 10.1186/s13063-017-2094-1 (PMC5526285; doi:10.1186/s13063-017-2094-1)
Supplement: Supplementary file 3 — Comparison of baseline demographics in study completers and dropouts. (DOCX 17 kb) [file 13063_2017_2094_MOESM3_ESM.docx]

Table S3

*Comparison of Baseline Demographics in Study Completers and Dropouts*

|  | Completers  (*n=* 126) | Dropouts  (*n=* 9) |  |  |  |
| --- | --- | --- | --- | --- | --- |
|  | Mean (+ SD) | | *t* | *df* | *p* |
| Age (years) | 45.31 (11.28) | 42.67 (13.20) | .67 | 132 | .50 |
|  | % | | *χ^2^* | *df* | *p* |
| Gender |  |  | .69 | 1 | .41 |
| Female | 76.98 | 88.89 |  |  |  |
| Male | 23.02 | 11.11 |  |  |  |
| Ethnicity^a^ |  |  | .69 | 1 | .41 |
| Caucasian | 92.86 | 100 |  |  |  |
| Other^b^ | 7.14 | 0 |  |  |  |
| Marital status^a^ |  |  | 1.25 | 2 | .54 |
| Married/Common-law | 72.22 | 55.56 |  |  |  |
| Divorced/Seperated | 11.90 | 22.22 |  |  |  |
| Never Married | 15.87 | 22.22 |  |  |  |
| Education Level^a^ |  |  | 1.10 | 3 | .78 |
| Completed High School | 11.11 | 22.22 |  |  |  |
| Some University | 24.60 | 22.22 |  |  |  |
| Completed University | 46.83 | 44.44 |  |  |  |
| Completed Graduate School | 17.46 | 11.11 |  |  |  |
| Annual Family Income^a^ ($) |  |  | 7.49 | 3 | .06 |
| 20,000-39,999 | 6.35 | 33.33 |  |  |  |
| 40,000-59,999 | 7.14 | 11.11 |  |  |  |
| 60,000-79,999 | 8.73 | 11.11 |  |  |  |
| >80,000 | 44.44 | 22.22 |  |  |  |
| Employment Status^a^ |  |  | 5.56 | 4 | .23 |
| Full time | 69.84 | 44.44 |  |  |  |
| Part time | 11.11 | 33.33 |  |  |  |
| Homemaker | 7.14 | 0 |  |  |  |
| Retired | 7.14 | 11.11 |  |  |  |
| Unemployed | 4.76 | 11.11 |  |  |  |
| *Note.* ^a^ Some cells had an expected count less than 5, so a Fisher’s exact test was selected. ^b^ Due to low numbers in the different ethnicity cells, they were collapsed in the “other group,” which consisted of Asian, East Indian, and Hispanic. | | | | | |
