# Supplementary material for: Behavioral weight-loss treatment plus motivational interviewing versus attention control: lessons learned from a randomized controlled trial
Source: Trials. 2017 Jul 25;18:351. doi: 10.1186/s13063-017-2094-1 (PMC5526285; doi:10.1186/s13063-017-2094-1)
Supplement: Supplementary file 4 — Comparison between study completers and dropouts on primary and exploratory outcome variables at baseline. (DOCX 16 kb) [file 13063_2017_2094_MOESM4_ESM.docx]

Table S4

*Comparison between Study Completers and Dropouts on Primary and Exploratory Outcome Variables at Baseline*

|  | Means (+ SD) | | *t* | *df* | *p* |
| --- | --- | --- | --- | --- | --- |
| Outcome measure | Study completers (*n=* 126) | Dropouts  (*n=* 9) |  |  |  |
| Weight (kg)^a^ | 91.37 (18.01) | 102.26 (29.90) | 1.08 | 8.42 | .31 |
| Weight change ratings |  |  |  |  |  |
| Importance | 8.92 (1.31) | 9.22 (1.09) | .90 | 104 | .37 |
| Readiness | 8.73 (1.38) | 9.00 (1.07) | .72 | 103 | .47 |
| Confidence | 7.96 (1.71) | 8.00 (1.63) | .35 | 102 | .73 |

*Note.* ^a^Equal variances were not assumed for this comparison.
