# Supplementary material for: Behavioral weight-loss treatment plus motivational interviewing versus attention control: lessons learned from a randomized controlled trial
Source: Trials. 2017 Jul 25;18:351. doi: 10.1186/s13063-017-2094-1 (PMC5526285; doi:10.1186/s13063-017-2094-1)
Supplement: Supplementary file 5 — Additional secondary outcome variables and associated results. A list of additional secondary outcome variables used to provide additional indicators of health, and their associated data analyses and results. (DOCX 42 kb) [file 13063_2017_2094_MOESM5_ESM.docx]

**Additional Secondary Outcome Variables and Associated Results**

**Outcome Variables**

**Body Mass Index (BMI).**

Body Mass Index was calculated as measured weight (in kilograms) divided by the square of height (in meters).

**Physical activity.**

The Paffenbarger questionnaire (PPAQ) assesses amount of activity performed during a typical week, and is validated to assess planned weekly and lifestyle associated physical activity [1]. The PPAQ consists of three major components: (1) stair climbing, (2) walking, and (3) sports and recreation. Participants are asked to report the frequency and duration of physical activity in the past week. Reported sports and recreational activities were coded by a research assistant, blind to study hypotheses and participant’s treatment condition, into three levels of intensity according to the PPAQ coding scheme: (1) light intensity (5 kcal/min), (2) moderate intensity (7.5 kcal/min), and (3) vigorous intensity (10 kcal/min). Scoring yields energy expenditure from physical activity per week (kcal/kg/week).

**Dietary behaviour.**

The Fat-related Dietary Habits Questionnaire (DHQ)[[2](#_ENREF_2)] is a 22-item questionnaire that was used to collect information on dietary behaviours and high-fat eating patterns. The DHQ consists of an overall summary score and five subscale scores assessing different dimensions fat-related dietary habits: 1) substitution (i.e., frequency of substituting fat-modified foods for high-fat foods); 2) modify meat (i.e., frequency of modifying meat products, such as removing skin, to be lower fat); 3) avoid frying; 4) replacement (i.e., replacing high fat foods with fruits and vegetables); and 5) avoid fat (i.e, avoiding use of fat, such as butter, as a flavouring). Responses are scored on a 4-point scale (usually, often, sometimes, rarely/never). Subscale scores are calculated as the mean of non-missing items for each subscale, and higher scores correspond to higher fat intakes. The overall summary score, the mean of all non-missing subscale scores, was calculated and used in analyses. A one-unit decrease in the summary score corresponds to a 13% decrease in total percent of energy from fat.

**Blood pressure.**

A measure of systolic and diastolic blood pressure was taken in a standardized manner according to the Canadian Hypertension Education Program Guidelines [[3](#_ENREF_3)]. Three blood pressure readings were obtained at each assessment time point.

**Eating disorder psychopathology.**

The Eating Disorder Examination Questionnaire (EDE-Q)[[4](#_ENREF_4)] was used to assess for eating disorder psychopathology. A questionnaire was used in addition to the SCID interview to obtain a quantitative measure of eating disorder symptoms and to examine the impact of the intervention on such symptoms. The EDE-Q is a 36-item self-report rating scale, which assesses the presence and degree of specific psychopathology associated with eating disorders over the previous 28 days. The EDE-Q consists of a global score as well as four subscales: Eating Concern, Restraint, Shape Concern, and Weight Concern [[5](#_ENREF_5)]. Higher scores are indicative of greater eating disorder psychopathology. The EDE-Q subscales show excellent internal consistency, with Cronbach alpha coefficients ranging from .78 to .93 [[6](#_ENREF_6)]. In the current study, alpha coefficients across the four assessments ranged from .53 to .78 for Restraint, .71 to .80 for Eating Concern, .86 to .91 for Shape Concern, and .67 to .77 for Weight Concern.

**Data Analysis**

**BMI, physical activity, and dietary behaviour.**

Linear mixed modeling (LMM) was used to test the effects of motivational interviewing on BMI, physical activity (total kilocalories/week), and dietary behaviour (overall dietary summary score). A two-level LMM design was employed, with the repeated time measurements set as the lowest level of analysis (Level 1), and the cases (i.e., participants) set as the grouping variable (Level 2). All statistical models included the predictor variables: baseline value of outcome variable, treatment group, and time. Gender effects were also examined for the primary and secondary outcomes variables by adding the variable ‘gender’ to the above specified model.

Given that the observed data showed non-linear trends for all outcome variables, longitudinal trends for each outcome variable were modeled using time as a categorical variable. Initially the potential interaction between group and time was examined by entering the variables: group, time, baseline value of outcome variable and the interaction term between time and group. In this model, the interaction term was of primary interest to determine if the intervention effect varied between groups. If the interaction term was non-significant, the model was subsequently refitted with only the main effect terms.

An alpha of *p* < .05 was used for evaluating statistical significance. For each time contrast in the main effect model, 95% confidence intervals (CI) were calculated. The structure of the repeated measures was modeled by including intercept as a random effect at the subject level.

**Blood pressure.**

LMM was also implemented to test the effects of MI on blood pressure. For the blood pressure data, paired sample t-tests were used to compare the first reading to the second and the third reading taken at each assessment. Given that no significant differences emerged among the three readings, we averaged them to obtain one reading for each assessment time point. For blood pressure, an identical modeling procedure was used as that utilized for the variables listed above. Statistical models included the predictor variables: baseline value of outcome variable, treatment group, and time.

**Eating disorder psychopathology.**

LMM was also implemented to examine the impact of the MI intervention on eating disorder psychopathology (EDE-Q data). An identical procedure was used as that utilized for the variables above. Statistical models included the predictor variables: baseline value of outcome variable, treatment group, and time.

**Results**

**BMI**

The interaction model for BMI was not significant. For the main effects model of BMI, the main effect of group was not significant, *F*(1, 138.76) = 1.27, *p* = .26. However, the main effect of time was significant, *F*(2, 261.68) = 20.41, *p* < .001. For both groups, BMI decreased significantly from baseline to the end of the TrymGym program, *B* = -.90, *SE* = .14; *t*(262.64) = -6.33, *p* < .001 (95% CI = -1.18, -.62; ES = .14). BMI also decreased significantly from baseline to the 6 month follow-up^[[1]](#footnote-1)^, *B* = -.32, *SE* = .14; *t*(261.68) = -2.25, *p* = .03 (CI = -.60, -.04; ES = .05). BMI means and standard errors for both groups across assessment time points are presented in Supplementary Table 1 below.

**Physical activity (kcal/kg/week)**

The interaction model for physical activity was not significant. As the physical activity outcome variable violated the assumption of normality (Kolmogorov-Smirnov *z* = 2.48, *p* < .001), a square root transformation was performed. As the model results did not substantially differ regardless of whether or not the variable was transformed, findings from the untransformed model are presented to assist with interpretation. An interaction model was first run for physical activity. For the main effects model, the main effect of group was not significant, *F*(1, 133.25) = .23, *p* = .63. However, the main effect of time was significant, *F*(3, 346.85) = 12.35, *p* < .001. For both groups, physical activity increased significantly from baseline to the end of the TrymGym program, *B* = 803.81, *SE* = 159.48; *t*(346.53) = 5.04, *p* < .001 (CI = 490.81, 1118.14; ES = .66). Physical activity also increased significantly from baseline to the 1 month follow-up, *B* = 863.82, *SE* = 162.27; *t*(349.93) = 5.32, *p* < .001 (CI = 545.27, 1183.56; ES = .71), and the 6 month follow-up, *B* = 549.75, *SE* = 346.19; *t*(346.19) = 3.45, *p* < .001 (CI = 237.04, 864.28; ES = .45). Physical activity means and standard errors for both groups across assessment time points are presented in Supplementary Table 2 below.

**Dietary behaviour (DHQ overall summary score)**

The interaction model for dietary behaviour was not significant. For the main effects model of the dietary global score, the main effect of group was not significant, *F*(1, 131.69) = 1.81, *p* = .18. However, the main effect of time was significant, *F*(3, 347.55) = 14.28, *p* < .001. For both groups, the dietary global score decreased significantly from baseline to the end of the TrymGym program, *B* = -.21, *SE* = .03; *t*(346.11) = -6.28, *p* < .001 (CI = -.28, -.15; ES = .46). The dietary global score also decreased significantly from baseline to the 1 month follow-up, *B* = -.15, *SE* = .03; *t*(350.91) = -4.39, *p* < .001 (CI = -.22, -.09; ES = .33), and the 6 month follow-up, *B* = -.10, *SE* = .03; *t*(347.10) = -2.84, *p* = .005 (CI = -.17, -.03; ES = .22). Means and standard errors for dietary behaviour across assessment time points are presented in Supplementary Table 2 below.

**Blood pressure**

**Systolic blood pressure.**

The interaction model for systolic blood pressure was not significant. As the systolic blood pressure variable violated the assumption of normality (Kolmogorov-Smirnov *z* = 1.62, *p* = .01), a log transformation was performed. As the model results did not substantially differ regardless of whether or not the variable was transformed, findings from the untransformed model are presented to assist with interpretation. First, an interaction model was run for systolic blood pressure, which included the group by time interaction. For the main effects model, the main effect of group was not significant, *F*(1, 131.94 = .10, *p* = .75. Similarly, the main effect of time also was not significant, *F*(2, 253.24) = .90, *p* = .41.

**Diastolic blood pressure.**

The interaction model for diastolic blood pressure was not significant. For the main effects model for diastolic blood pressure, the main effect of group was not significant, *F*(1, 131.12 = 1.00, *p* = .32. Similarly, the main effect of time also was not significant, *F*(2, 253.09) = .90, *p* = .15. Means and standards errors for systolic and diastolic blood pressure across assessment time points are presented in Supplementary Table 3 below.

**Eating disorder psychopathology**

**EDE-Q Global Score.**

All of the interaction models for EDE-Q outcomes were not significant. For the main effects model of EDE-Q global score, the main effect of group was not significant, *F*(1, 128.41) = .49, *p* = .49. However, the main effect of time was significant, *F*(3, 328.17) = 9.28, *p* < .001. For both groups, the EDE-Q global score decreased significantly from baseline to the end of the TrymGym program, *B* = -.23, *SE* = .07; *t*(328.78) = -3.43, *p* = .001 (CI = -.36, -.10; ES = .23). The global score also decreased significantly from baseline to the 1 month follow-up, *B* = -.26, *SE* = .07; *t*(330.55) = -3.80, *p* < .001 (CI = -.39, -.13; ES = .26), and the 6 month follow-up, *B* = -.33, *SE* = .07; *t*(328.02) = -4.89, *p* < .001 (CI = -.46, -.20; ES = .33). Means and standard errors for eating disorder psychopathology (i.e., the EDE-Q global score and subscale scores) across assessment time points are presented in Supplementary Table 4 below.

**EDE-Q Restraint subscale.**

For the main effects model of the EDE-Q restraint subscale, the main effect of group was not significant, *F*(1, 128.20) = 1.95, *p* = .17. However, the main effect of time was significant, *F*(3, 336.51) = 14.32, *p* < .001. For both groups, the restraint subscale increased significantly from baseline to the end of the TrymGym program, *B* = .44, *SE* = .12; *t*(336.42) = 3.89, *p* < .001 (CI = .22, .68; ES = .36). The restraint subscale did not significantly change from baseline to the 1 month follow-up, *B* = .20, *SE* = .12; *t*(338.77) = 1.69, *p* = .09 (CI = -.03, .43; ES = .16). Then, at the 6 month follow-up, the restraint subscale decreased significantly relative to baseline, *B* = -.31, *SE* = .12; *t*(355.59) = -2.73, *p* = .007 (CI = -.54, -.09; ES = .25).

**EDE-Q Shape concern subscale.**

For the main effects model for the EDE-Q shape concern subscale, the main effect of group was not significant, *F*(1, 129.77) = .24, *p* = .64. However, the main effect of time was significant, *F*(3, 334.79) = 23.37, *p* < .001. For both groups, the shape concern subscale decreased significantly from baseline to the end of the TrymGym program, *B* = -.73, *SE* = .10; *t*(335.28) = -7.12, *p* < .001 (CI = -.53, -.92; ES = .48) The shape concern subscale also decreased significantly from baseline to the 1 month follow-up, *B* = -.73, *SE* = .10; *t*(337.39) = -7.06, *p* < .001 (CI = -.93, -.52; ES = .48), and the 6 month follow-up, *B* = -.53, *SE* = .10; *t*(334.52) = -5.21, *p* < .001 (CI = -.73, -.33; ES = .35).

**EDE-Q Weight concern subscale.**

For the main effects model of the EDE-Q weight concern subscale, the main effect of group was not significant, *F*(1, 130.16) = .86, *p* = .36. However, the main effect of time was significant, *F*(3, 335.39) = 19.47, *p* < .001. For both groups, the weight concern subscale decreased significantly from baseline to the end of the TrymGym program, *B* = -.52, *SE* = .09; *t*(335.83) = -6.02, *p* < .001 (CI = -.69, -.35; ES = .44). The weight concern subscale also decreased significantly from baseline to the 1 month follow-up, *B* = -.55, *SE* = .09; *t*(338.00) = -6.31, *p* < .001 (CI = -.72, -.38; ES = .47), and the 6 month follow-up, *B* = -.51, *SE* = .09; *t*(335.05) = -5.91, *p* < .001 (CI = -.68, -.34; ES = .43).

**EDE-Q Eating concern.**

As the eating concern subscale violated the assumption of normality (Kolmogorov-Smirnov *z* = 3.18, *p* < .001), an inverse transformation was performed. As the model results did not substantially differ regardless of whether or not the variable was transformed, findings from the untransformed model are presented to assist with interpretation (see Appendix L for results for the inverse transformed variable). For the main effects model, the main effect of group was not significant, *F*(1, 128.06) = .43, *p* = .52, nor was the main effect of time, *F*(3, 334.15) = 1.75, *p* = .16.

References

1. Paffenbarger RS, Wing AL, Hyde RT. Physical activity as an index of heart attack risk in college alumni. American Journal of epidemiology. 1978;108(3):161-75.

2. Kristal AR, Shattuck AL, Henry HJ. Patterns of dietary behavior associated with selecting diets low in fat: reliability and validity of a behavioral approach to dietary assessment. J Am Diet Assoc. 1990;90(2):214-20.

3. Hemmelgarn BR, McAlister FA, Grover S, Myers MG, McKay DW, Bolli P, et al. The 2006 Canadian Hypertension Education Program recommendations for the management of hypertension: Part I–Blood pressure measurement, diagnosis and assessment of risk. Canadian Journal of Cardiology. 2006;22(7):573-81.

4. Fairburn CG, Beglin SJ. Assessment of eating disorders: Interview or self‐report questionnaire? International journal of eating disorders. 1994;16(4):363-70.

5. Cooper Z, Cooper PJ, Fairburn CG. The validity of the eating disorder examination and its subscales. The British Journal of Psychiatry. 1989;154(6):807-12.

6. Luce KH, Crowther JH. The reliability of the eating disorder examination—Self‐report questionnaire version (EDE‐Q). International Journal of Eating Disorders. 1999;25(3):349-51.

Table S1

*Means and Standard Errors for MI and Control Groups on Body Mass Index*

|  | Baseline | |  | End of BWLP^a^ | |  |  |  | 6 month follow-up^a^ | |  |
| --- | --- | --- | --- | --- | --- | --- | --- | --- | --- | --- | --- |
| Outcome measure | *M* | *SE* |  | *M* | *SE* | ES |  |  | *M* | *SE* | ES |
| Body Mass Index |  |  |  |  |  |  |  |  |  |  |  |
| MI Group | 33.78 | .72 |  | 32.27 | .16 | +.048 |  |  | 32.96 | .16 | +.005 |
| Control Group | 33.37 | .81 |  | 32.64 | .16 |  |  |  | 33.11 | .16 |  |
| *Note.* The unit of measurement for body mass index is weight in kilograms divided by height squared. MI = motivational interviewing; BWLP = behavioural weight loss program; ^a^ Mean values are calculated with baseline value as covariate. MLM ES (effect size) calculated as d_GMA-RAW_ = estimated coefficient(time)/SD_RAW_ (Feingold, 2009). (+) ES favors MI group; (-) ES favors control group. | | | | | | | | | | | |

Table S2

*Means and Standard Errors for MI and Control Groups on Physical Activity and Dietary Behaviour*

|  | Baseline | |  | End of BWLP^a^ | |  |  | 1 month follow-up^a^ | |  |  | 6 month follow-up^a^ | |  |
| --- | --- | --- | --- | --- | --- | --- | --- | --- | --- | --- | --- | --- | --- | --- |
| Outcome measure | *M* | *SE* |  | *M* | *SE* | ES |  | *M* | *SE* | ES |  | *M* | *SE* | ES |
| PPAQ |  |  |  |  |  |  |  |  |  |  |  |  |  |  |
| MI Group | 1574.46 | 156.92 |  | 2161.80 | 179.58 | -.135 |  | 2196.78 | 184.44 | -.191 |  | 2060.09 | 178.15 | -.208 |
| Control Group | 1183.37 | 139.46 |  | 2211.87 | 186.56 |  |  | 2299.22 | 191.69 |  |  | 1789.34 | 187.92 |  |
| DHQ overall |  |  |  |  |  |  |  |  |  |  |  |  |  |  |
| MI Group | 2.90 | .06 |  | 2.64 | .04 | +.078 |  | 2.71 | .04 | +.016 |  | 2.80 | .04 | +.142 |
| Control Group | 2.78 | .06 |  | 2.62 | .04 |  |  | 2.67 | .04 |  |  | 2.70 | .04 |  |
| *Note.* The unit of measurement for physical activity is kilocalories per week. MI = motivational interviewing; BWLP = behavioural weight loss program; PPAQ = Paffenbarger Physical Activity Questionnaire. DHQ overall = Fat-related Dietary Habits Questionnaire overall summary score. ^a^ Mean values are calculated with baseline value as covariate. MLM ES (effect size) calculated as *d*_GMA-RAW_ = estimated coefficient(time)/SD_RAW_ (Feingold, 2009). (+) ES favors MI group; (-) ES favors control group. | | | | | | | | | | | | | | |

Table S3

*Means and Standard Errors for MI and Control Groups on Blood Pressure Outcomes*

|  | Baseline | |  | End of BWLP^a^ | |  |  | 6 month follow-up^a^ | |  |
| --- | --- | --- | --- | --- | --- | --- | --- | --- | --- | --- |
| Outcome measure | *M* | *SE* |  | *M* | *SE* | ES |  | *M* | *SE* | ES |
| Blood Pressure Systolic (mmHg) |  |  |  |  |  |  |  |  |  |  |
| MI Group | 128.62 | 1.64 |  | 127.84 | 1.03 | -.038 |  | 128.93 | 1.03 | -.005 |
| Control Group | 128.51 | 1.68 |  | 127.19 | 1.05 |  |  | 128.67 | 1.09 |  |
| Blood Pressure Diastolic (mmHg) |  |  |  |  |  |  |  |  |  |  |
| MI Group | 78.46 | 1.18 |  | 77.48 | .68 | +.014 |  | 76.77 | .69 | +.065 |
| Control Group | 77.89 | 1.33 |  | 77.89 | .68 |  |  | 77.82 | .72 |  |
| *Note.* MI = motivational interviewing; BWLP = behavioural weight loss program. ^a^ Mean values are calculated with baseline value as covariate. MLM ES (effect size) calculated as *d*_GMA-RAW_ = estimated coefficient(time)/SD_RAW_ (Feingold, 2009). (+) ES favors MI group; (-) ES favors control group. | | | | | | | | | | |

Table S4

*Means and Standard Errors for MI and Control Groups on Eating Disorder Psychopathology*

|  | Baseline | |  | End of BWLP^a^ | |  |  | 1 month follow-up^a^ | |  |  | 6 mont follow-up^a^ | |  |
| --- | --- | --- | --- | --- | --- | --- | --- | --- | --- | --- | --- | --- | --- | --- |
| Outcome measure | *M* | *SE* |  | *M* | *SE* | ES |  | *M* | *SE* | ES |  | *M* | *SE* | ES |
| EDE-Q |  |  |  |  |  |  |  |  |  |  |  |  |  |  |
| Global Score |  |  |  |  |  |  |  |  |  |  |  |  |  |  |
| MI Group | 2.27 | .14 |  | 2.01 | .08 | -.002 |  | 1.91 | .08 | +.214 |  | 1.96 | .08 | -.135 |
| Control Group | 2.34 | .11 |  | 2.07 | .08 |  |  | 2.13 | .09 |  |  | 1.92 | .08 |  |
| Restraint |  |  |  |  |  |  |  |  |  |  |  |  |  |  |
| MI Group | 1.88 | .16 |  | 2.39 | .13 | +.096 |  | 2.01 | .14 | -.185 |  | 1.58 | .13 | -.005 |
| Control Group | 2.10 | .15 |  | 2.47 | .14 |  |  | 2.35 | .14 |  |  | 1.75 | .14 |  |
| Eating Concern |  |  |  |  |  |  |  |  |  |  |  |  |  |  |
| MI Group | 1.12 | .17 |  | .89 | .09 | +.018 |  | 1.00 | .09 | +.098 |  | 1.09 | .09 | -.109 |
| Control Group | 0.99 | .13 |  | .82 | .10 |  |  | 1.03 | .10 |  |  | .94 | .10 |  |
| Weight Concern |  |  |  |  |  |  |  |  |  |  |  |  |  |  |
| MI Group | 2.89 | .15 |  | 2.28 | .11 | +.089 |  | 2.24 | .11 | +.116 |  | 2.37 | .10 | -.081 |
| Control Group | 2.92 | .14 |  | 2.46 | .11 |  |  | 2.44 | .11 |  |  | 2.39 | .11 |  |
| Shape Concern |  |  |  |  |  |  |  |  |  |  |  |  |  |  |
| MI Group | 3.25 | .20 |  | 2.54 | .13 | -.097 |  | 2.43 | .13 | +.194 |  | 2.81 | .12 | -.144 |
| Control Group | 3.39 | .19 |  | 2.61 | .13 |  |  | 2.72 | .13 |  |  | 2.72 | .13 |  |
| *Note.* MI = motivational interviewing; BWLP = behavioural weight loss program; EDE-Q = Eating Disorder Examination Questionnaire. ^a^ Mean values are calculated with baseline value as covariate. MLM ES (effect size) calculated as *d*_GMA-RAW_ = estimated coefficient(time)/SD_RAW_ (Feingold, 2009). (+) ES favors MI group; (-) ES favors control group. | | | | | | | | | | | | | | |

1. At 6 month follow-up, note there is a discrepancy between BMI and weight results. Although BMI (kg/m^2^) decreased significantly from baseline, weight (kg) did not decrease significantly. This discrepancy reflects differences in effect sizes, both of which were small. The magnitude of BMI change was small from baseline to the 6 month follow-up (ES = .05), as was the magnitude of weight change during the same frame (ES = .03). In sum, although the BWLP treatment resulted in small reductions in both BMI and weight from baseline to the end of the program, only BMI change between baseline and 6 month follow-up reached statistical significance. [↑](#footnote-ref-1)
